# Predicting the risk of postoperative recurrence and high-grade histology in patients with intracranial meningiomas using routine preoperative MRI

**DOI:** 10.1007/s10143-020-01301-7

**Published:** 2020-04-23

**Authors:** Dorothee Cäcilia Spille, Alborz Adeli, Peter B. Sporns, Katharina Heß, Eileen Maria Susanne Streckert, Caroline Brokinkel, Christian Mawrin, Werner Paulus, Walter Stummer, Benjamin Brokinkel

**Affiliations:** 1 Department of Neurosurgery, University Hospital Münster, Münster, Albert-Schweitzer-Campus 1, Building A1, 48149 Münster, North Rhine Westphalia Germany; 2 Institute of Clinical Radiology, University of Münster, Münster, North Rhine Westphalia Germany; 3 Institute of Neuropathology, University Hospital Münster, Münster, North Rhine Westphalia Germany; 4 Institute of Neuropathology, Otto-von-Guericke University, Magdeburg, Saxony-Anhalt Germany

**Keywords:** Magnetic resonance imaging, Meningiomas, MRI, Recurrence, WHO grade

## Abstract

Risk factors for prediction of prognosis in meningiomas derivable from routine preoperative magnetic resonance imaging (pMRI) remain elusive. Correlations of tumor and edema volume, disruption of the arachnoid layer, heterogeneity of contrast enhancement, enhancement of the capsule, T2-intensity, tumor shape, and calcifications on pMRI with tumor recurrence and high-grade (WHO grade II/III) histology were analyzed in 565 patients who underwent surgery for WHO grade I (*N* = 516, 91%) or II/III (high-grade histology, *N* = 49, 9%) meningioma between 1991 and 2018. Edema volume (OR, 1.00; *p* = 0.003), heterogeneous contrast enhancement (OR, 3.10; *p* < 0.001), and an irregular shape (OR, 2.16; *p* = 0.015) were associated with high-grade histology. Multivariate analyses confirmed edema volume (OR, 1.00; *p* = 0.037) and heterogeneous contrast enhancement (OR, 2.51; *p* = 0.014) as risk factors for high-grade histology. Tumor volume (HR, 1.01; *p* = 0.045), disruption of the arachnoid layer (HR, 2.50; *p* = 0.003), heterogeneous contrast enhancement (HR, 2.05; *p* = 0.007), and an irregular tumor shape (HR, 2.57; *p* = 0.001) were correlated with recurrence. Multivariate analyses confirmed tumor volume (HR, 1.01; *p* = 0.032) and disruption of the arachnoid layer (HR, 2.44; *p* = 0.013) as risk factors for recurrence, independent of histology. Subgroup analyses revealed disruption of the arachnoid layer (HR, 9.41; *p* < 0.001) as a stronger risk factor for recurrence than high-grade histology (HR, 5.15; *p* = 0.001). Routine pMRI contains relevant information about the risk of recurrence or high-grade histology of meningioma patients. Loss of integrity of the arachnoid layer on MRI had a higher prognostic value than the WHO grading, and underlying histological or molecular alterations remain to be determined.

**Electronic supplementary material:**

The online version of this article (10.1007/s10143-020-01301-7) contains supplementary material, which is available to authorized users.

## Introduction

Meningiomas are the most common primary intracranial neoplasms and usually treated by surgical resection and/or irradiation followed by radiological surveillance [5]. Although surgery is generally considered to provide local tumor control, rates of progression strongly depend on the extent of resection, and recurrence has been reported in up to 20% even after gross total removal [6, 24, 28, 32]. Hence, estimation of the risk of postoperative tumor recurrence remains crucial during care of meningioma patients.

Despite increasing knowledge about several molecular alterations, such as loss of H3K27M trimethylation or hTERT promoter mutations, being associated with prognosis [13, 25, 30], estimation of the risk of tumor recurrence in daily clinical routine usually refers to the extent of resection and the WHO grade of the tumor [5, 6, 24, 28]. Several retrospective studies suggested correlations between findings on preoperative magnetic resonance imaging (MRI), e.g., edema formation [11, 21], tumor volume [2, 10], disruption of the arachnoid layer [17, 18, 20, 21] or lobulated growth [7, 14], and postoperative tumor recurrence or high-grade (WHO grade II/III) histology [29]. However, the results were mostly obtained from analyses of smaller cohorts and were partially inconsistent. In addition, radiological findings found to be associated with high-grade histology in some studies were not necessarily correlated with prognosis, underlining the importance of further elucidation.

Considering the availability of preoperative MRI sequences (e.g. ,T1-weighted contrast-enhanced, T2-weighted) in the vast majority of meningioma patients [27], identification of risk factors for recurrence on routine preoperative imaging could improve the estimation of prognosis and might subsequently impact postoperative surveillance. In this study, we therefore investigated correlations of MRI characteristics with postoperative recurrence or high-grade histology in a series of > 550 meningiomas.

## Materials and methods

### Data collection

Patients were retrieved from the local department meningioma database containing all meningioma surgeries performed between 1991 and 2018. Of 1306 surgeries, 565 patients with (a) initially diagnosed meningioma, (b) intracranial tumor location, (c) available follow-up data, and (d) preoperative MRI were identified and subjected to further statistical analyses. Medical records and operative reports of all patients who underwent surgery for intracranial meningioma in our department between 1991 and 2018 were reviewed. Medical data subjected to statistical analyses included patient’s sex and age at the time of surgery, the extent of resection classified intraoperatively by the neurosurgeon as gross total resection, GTR (Simpson grades I-II) and subtotal resection, STR (Simpson grades III-V [28]), and the administration of postoperative irradiation. In general, adjuvant irradiation was administered for primary diagnosed grade III and recurrent or subtotally resected grade II tumors as well as in benign lesions following debulking [5]. No chemotherapy was administered. As it is standard in our institution, follow-up contrast-enhanced imaging was performed 3 and 6 months after surgery and was then repeated annually and semi-annually in patients with benign and high-grade meningiomas, respectively. Imaging was evaluated for tumor progression by a team of two independent observers (at least one neurosurgeon and one (neuro-)radiologist). Progression was diagnosed in case of any detected tumor growth on MRI/CT (computed tomography) beyond technical-dependent measurement range, new affection of the adjacent brain tissue, detected by edema formation, or new clinical symptoms, with or without subsequent indication for further surgical treatment. Contrast-enhanced CT scans were performed in case of any contraindications for MRI. Follow-up regarding tumor progression was updated using standardized questionnaires which were sent to the primary care takers. Representative tumor specimen from each surgery were neuropathologically reviewed and diagnosed according to the 2016 WHO Classification of Central Nervous System Tumors [24].

### Radiological data

**Fig. 1:**
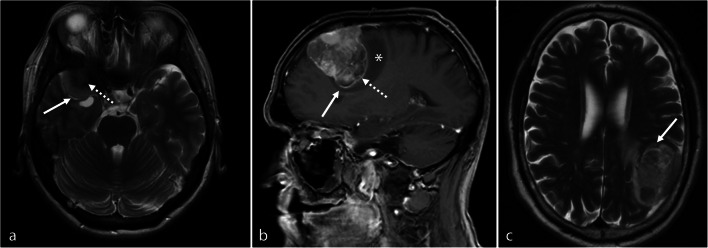
Illustrative samples of the analyzed radiological variables. In **a**, axial T2-weighted MRI shows a thin cerebrospinal fluid cleft (solid arrow, intact arachnoid layer) at the surface between the brain and the T2-hyperintense, regular shaped tumor with some calcifications (dashed arrow) at its origin at the sphenoid ridge. In **b**, sagittal T1-weighted contrast-enhanced imaging shows an irregularly, mushroom-like shaped lesion (solid arrow) with heterogeneous gadolinium enhancement of the tumor, an enhancing tumor capsule (dashed arrow) and a moderate perifocal edema (asterisk). In **c**, axial T2-weighted MRI depicts the lack of a cerebrospinal fluid cleft at the brain/tumor surface, indicating a disruption of the arachnoid layer

Preoperative radiological imaging was analyzed by a team of two independent and experienced radiologists (AA and PBS). For statistical analyses, tumor location was classified as “skull base” and “non-skull base” location, subsuming convexity, intraventricular, posterior fossa, and parasagittal lesions. According to previous analyses investigating correlations between MRI findings and high-grade histology or progression [1, 29], the following radiological variables were examined (illustrative samples in Fig. 1): Tumor and edema volumes (V_T_ and V_E_) were calculated according to the formula for a spheroid *V* = 4/3 × π × r1 × r2 × r3 (“*r*” is the tumor radius at the site of its largest extension in axial (r1), coronal (r2), and sagittal (r3) planes [1, 8]. Edema volume was finally calculated by subtraction of the tumor from the edema volume. Disruption of the arachnoid layer was analyzed on T2-weighted imaging and diagnosed in case of an indistinct tumor border and/or lack of a cerebrospinal fluid cleft at the brain/meningioma surface. Contrast enhancement of the tumor was investigated on T1-weigthed gadolinium-enhanced images and diagnosed as either heterogeneous or homogenous. Similarly, capsular contrast enhancement was identified on T1-weigthed gadolinium-enhanced images and described as present, if more than half of the tumor surface enhanced, otherwise as absent. Intensity of the tumor was analyzed on T2-weighted images and classified as hyper-, iso-, or hypointense compared to the gray matter, while calcifications were classified as present or absent. Contrast enhancement of the tumor capsule was dichotomously evaluated as absent or present on gadolinium-enhanced T1-weighted imaging. The tumor shape was classified as regular or irregular, e.g., in terms of mushroom-like growth. All variables were thoroughly radiologically analyzed and classified based on the individual evaluation of the two radiologists. Disagreement was solved by discussion.

### Statistical analyses

Calculations were performed using standard commercial statistic software (IBM SPSS Statistics, Version 24, IBM, Germany). Data are described by standard statistics, e.g., median and range and absolute and relative frequencies for continuous and categorical variables and compared by Mann-Whitney-U and Fishers exact test, respectively. Progression-free interval (PFI) was calculated from the date of surgery to the date of progression, or, in case of an event-free survival, until the date of last follow-up. No threshold period between discharge and outpatient follow-up was chosen. PFI was further estimated by Kaplan-Meier analyses and compared by log-rank tests. Multivariable analyses for tumor recurrence were performed using Mantel-Cox test and backward Wald logistic regression and characterized by hazard (HR) or odds ratios (OR), 95% confidence intervals (CI), and Wald-test *p* values. Age, sex (female (ref.) vs male), tumor location (skull base (ref.) vs non-skull base), the extent of resection (GTR (ref) vs STR), the WHO grade (grade I (ref.) vs high-grade), and, due to numerous intercorrelations (suppl. table), all investigated radiological variables were included into multivariate analyses. All reported *p* values are two-sided. A *p* value of < 0.05 was considered to be statistically significant throughout the entire analyses. Data collection and scientific use were approved by the local ethics committee (Münster 2007-420-f-S and Münster 2018-061-f-S) and in accordance with the 1964 Helsinki declaration and its later amendments or comparable ethical standards.

## Results

**Fig. 2:**
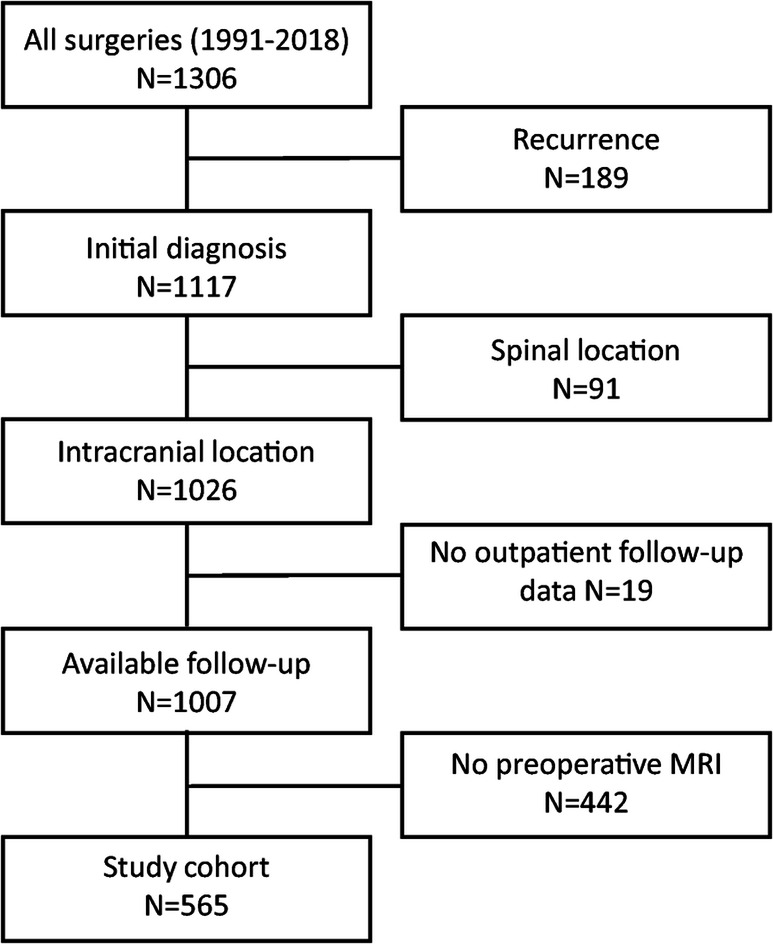
Consort diagram of patient selection. Of a total of 1306 surgeries between 1991 and 2018, 565 patients were subjected to statistical analyses

Using the above described approach, 565 patients (406 females, 72%; 159 males, 28%; median age 59 years, range: 7–91 years) with available preoperative MRI who underwent surgery between 1991 and 2018 were identified and included into subsequent analyses (Fig. 2). Data about adjuvant irradiation was available in 550 patients (97%); among those, adjuvant irradiation was administered in 42 cases (8%). Clinical, histopathological, and radiological data are summarized in Table 1. In univariate analyses, numerous correlations between the analyzed imaging variables were detected (suppl. table).

**Table 1:** Patient’s characteristics

Variable	Available data (*N*, *n*%)	Frequency/distribution (*N*, *n*%)
Age (median, range)	565 (100%)	59 years (7–91)
Sex	565 (100%)	
Males		159 (28%)
Females		406 (72%)
Location	565 (100%)	
Non-skull base		321 (57%)
Skull base		244 (43%)
Extent of resection	543 (96%)	
GTR		418 (77%)
STR		125 (23%)
WHO grade	565 (100%)	
I		516 (91%)
II/III		49 (9%)
Tumor volume (median, range)	504 (89%)	12.40 ccm (0.20–356.94)
Edema volume (median, range)	492 (87%)	0.00 ccm (0.00–739.28)
Intensity on T2-weighted MRI	501 (89%)	
Isointense		60 (3%)
Hypointense		270 (54%)
Hyperintense		215 (43%)
Arachnoid layer	489 (87%)	
Intact		208 (43%)
Disrupted		281 (57%)
Contrast enhancement	565 (100%)	
Homogeneous		326 (58%)
Heterogeneous		239 (42%)
Tumor shape	514 (91%)	
Regular		317 (62%)
Irregular		197 (38%)
Calcifications	500 (89%)	
Absent		395 (79%)
Present		105 (21%)
Capsular contrast enhancement	484 (86%)	
Absent		331 (68%)
Present		153 (32%)

Baseline clinical, histological, and radiological data were available in the vast majority of patients

### Routine preoperative MRI can distinctly improve prediction of high-grade histology

Associations between imaging variables and WHO grade are summarized in Table 2. Briefly, peritumoral edema volume (OR, 1.00; 95%CI 1.00–1.01; *p* = 0.003), heterogeneous contrast enhancement (OR, 3.10; 95%CI 1.67–5.78; *p* < 0.001), and an irregular tumor shape (OR, 2.16; 95%CI 1.16–4.00; *p* = .015) were correlated with high-grade histology in univariate analyses. Receiver operating characteristic (ROC) analyses revealed an optimal cut-off edema volume of 0.046 ccm for the prediction of high-grade histology (sensitivity 0.70; specificity 0.60; AUC, 0.645; *p* = 0.002). Similarly, risk of high-grade histology tended to be lower in tumors not arising from the convexity (OR, 0.55; 95%CI 0.29–1.04; *p* = 0.066). Multivariate analyses confirmed peritumoral edema volume (OR, 1.00, 95%CI 1.00–1.01; *p* = 0.037) and heterogeneous contrast enhancement (OR, 2.51; 95%CI 1.20–5.25; *p* = 0.014) as risk factors for WHO grade II/III histology.

**Table 2:** Correlations of clinical and radiological variables with high-grade histology in uni-and multivariate analyses

Variable	Univariate analysis: OR (95%CI), *p* value	Multivariate analysis: OR (95%CI), *p* value
Sex: male vs female (ref.)	3.57, 1.97–6.48; *p* < 0.001	2.39, 1.19–4.81; *p* = 0.014
Age at surgery (in years)	1.03, 1.01–1.05; *p* = 0.008	1.03, 1.01–1.06; *p* = 0.018
Tumor location: non-skull base vs skull base (ref.)	0.55, 0.29–1.04; *p* = 0.066	0.69, 0.32–1.50; *p* = 0.352
Tumor volume (in ccm)	1.01, 1.00–1.01; *p* = 0.175	1.00, 0.99–1.01, *p* = 0.792
Edema volume (in ccm)	1.00, 1.00–1.01; *p* = 0.003	1.00, 1.00–1.01; *p* = 0.037
Intensity on T2-weighted MRI		
Isointense vs hyperintense (ref.)	0.81, 0.18–3.75; *p* = 0.787	0.35, 0.06–1.95; *p* = 0.232
Hypointense vs hyperintense (ref.)	0.49, 0.10–2.36; *p* = 0.372	0.22, 0.04–1.28; *p* = 0.092
Arachnoid layer: interrupted vs intact (ref.)	1.66, 0.84–3.29; *p* = 0.146	1.32, 0.63–2.74; *p* = 0.461
Contrast enhancement: Heterogeneous vs Homogeneous (ref.)	3.10, 1.67–5.78; *p* < 0.001	2.51, 1.20–5.25; *p* = 0.014
Tumor shape: irregular vs regular (ref.)	2.16, 1.16–4.00; *p* = 0.015	1.09, 0.51–2.34; *p* = 0.818
Tumor calcifications: present vs absent (ref.)	1.33, 0.65–2.73; *p* = 0.442	0.84, 0.35–2.02; *p* = 0.694
Capsular contrast enhancement: present vs absent (ref.)	1.43, 0.74–2.76; *p* = 0.288	1.13, 0.53–2.43; *p* = 0.747

Several radiological and clinical variables were found to be associated with grade II/III histology

*OR* odds ratio, *CI* confidence interval, *ref.* reference

### Routine preoperative MRI can distinctly improve prediction of recurrence

**Fig. 3:**
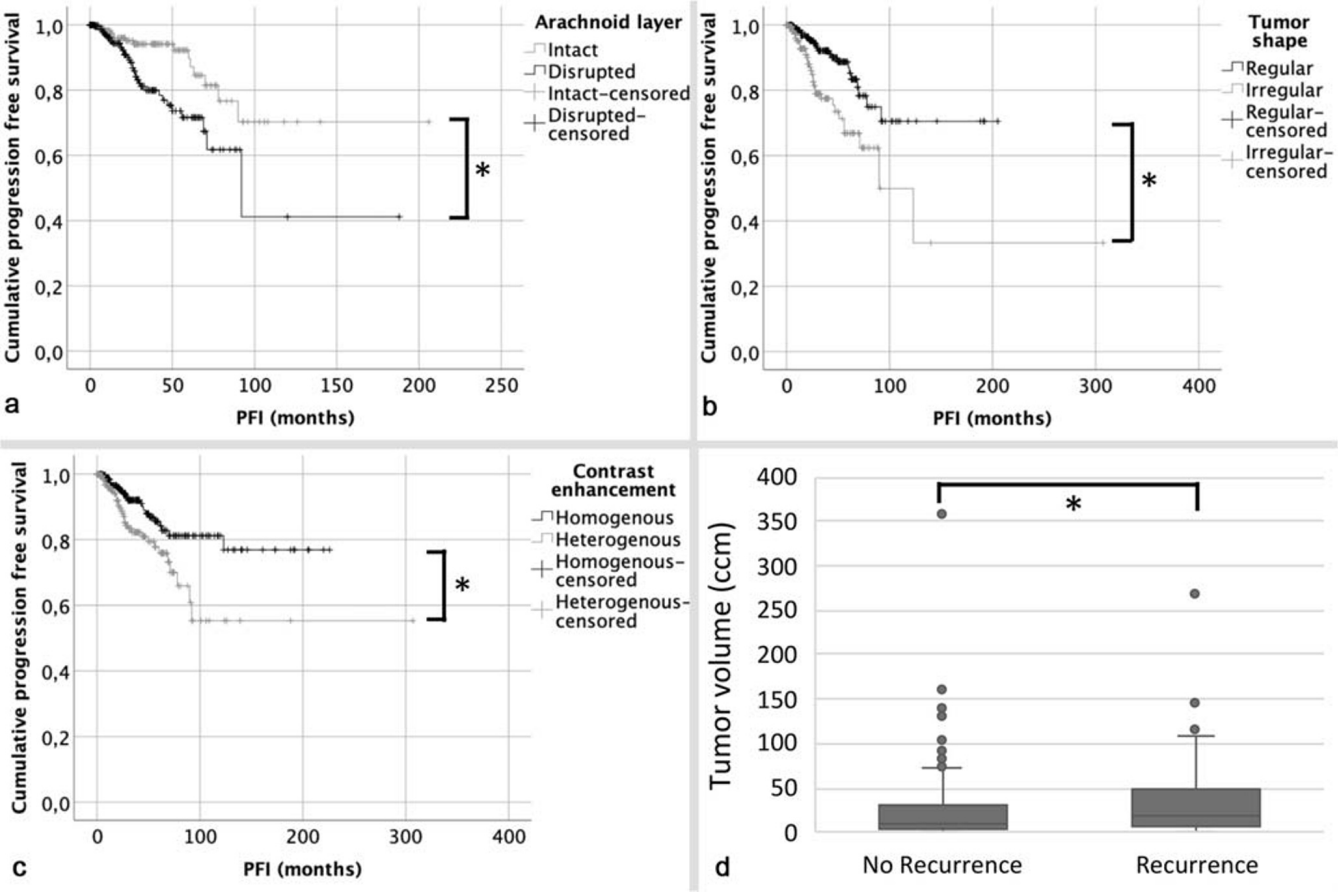
Kaplan-Meier (**a**–**c**) and Box plots (**d**) showing correlations between radiological variables and progression. Median PFI was 92 months and not reached in patients with an intact or disrupted arachnoid layer on the brain/tumor surface, respectively (**a**, *p* = 0.002, log-rank test). Similarly, median progression-free interval (PFI) was 90 months in individuals harboring irregularly shaped tumors, while median PFI was not reached in cases with regularly shaped meningiomas (**b**, *p* < 0.001). Median PFI significantly differed between patients with heterogeneous and homogeneous contrast enhancing tumors (**c**, *p* = 0.006, medians not reached). In **d**, Box and whiskers plots illustrate that the median tumor volume was higher in patients with than without developing recurrence during follow-up (18.72 ccm, range 0.70–267.77 ccm vs 10.71 ccm, range 0.02–356.94 ccm; *p* = 0.010). The boxes indicate upper and lower 25% quartile, the whiskers the minimum and maximum value, the dots the outliers, the asterisks the extreme values, and the heavy horizontal line indicates the median (*statistically significant, *ccm* cubic centimeter)

With a median follow-up of 26 months (range: 0–307 months), progression was observed in 58 cases (11%). In univariate analyses, risk of postoperative tumor recurrence was higher in males (HR, 2.10; 95%CI 1.25–3.54; *p* = 0.005), in high-grade meningiomas (HR, 4.69; 95%CI 2.72–8.07; *p* < 0.001), and, with borderline significance, after STR (HR, 1.82; 95%CI 1.00–3.32; *p* = 0.05). Among the analyzed radiological variables, disruption of the arachnoid layer (HR, 2.50; 95%CI 1.36–4.61; *p* = 0.003), heterogeneous contrast enhancement (HR, 2.05; 95%CI 1.22–3.46; *p* = 0.007), and an irregular, mushroom-like tumor shape (HR, 2.57; 95%CI 1.51–4.37; *p* = 0.001) were associated with an increased risk of recurrence (Table 3). Correspondingly, disruption of the arachnoid layer (Fig. 3a, *p* = 0.002), heterogeneous contrast enhancement (Fig. 3b, *p* = 0.006), and an irregular tumor shape (Fig. 3c, *p* < 0.001) were correlated with shorter PFI (see details in legend). Similarly, a rising tumor volume (HR, 1.01; 95%CI 1.00–1.01; *p* = 0.045) correlated with an increased risk of recurrence (Fig. 3d). ROC analyses revealed an optimal cut-off tumor volume of 11.32 ccm for the prediction of recurrence (sensitivity 0.65, specificity 0.51, AUC = 0.061; *p* = 0.010). Multivariate analyses adjusted for age, sex, WHO grade, and the analyzed radiological variables confirmed high-grade histology (HR, 4.58; 95%CI 2.41–8.71; *p* < 0.001), tumor volume (HR, 1.01; 95%CI 1.00–1.02; *p* = 0.032), and disruption of the arachnoid layer (HR, 2.44; 95%CI 1.21–4.92; *p* = 0.013) as risk factors for recurrence. No other correlations between any of the analyzed radiological variables and recurrence were found (Table 3).

**Table 3:** Correlations of clinical, histological, and radiological variables with recurrence in uni- and multivariate analyses

Variable	Univariate analysis: HR (95%CI), *p* value	Multivariate analysis: HR (95%CI), *p* value
Sex: male vs female (ref.)	2.10, 1.25–3.54; *p* = 0.005	1.29, 0.66–2.51; *p* = 0.460
Age at surgery (in years)	1.01, 0.99–103; *p* = 0.299	1.00, 0.98–1.03; *p* = 0.776
Subtotal resection vs gross total resection (ref.)	1.82, 1.00–3.32; *p* = 0.05	1.28, 0.63–2.60; *p* = 0.499
High-grade histology vs WHO grade I (ref.)	4.69, 2.72–8.07; *p* < 0.001	4.58, 2.41–8.71; *p* < 0.001
Tumor location: non-skull base vs skull base (ref.)	1.34, 0.83–2.31; *p* = 0.219	1.26, 0.66–2.43; *p* = 0.484
Tumor volume (in ccm)	1.01, 1.00–1.01; *p* = 0.045	1.01, 1.00–1.02; *p* = 0.032
Edema volume (in ccm)	1.00, 1.00–1.01; *p* = 0.110	1.00, 1.00–1.00; *p* = 0.490
Intensity on T2-weighted MRI		
Isointense vs hyperintense (ref.)	0.82, 0.25–2.73; *p* = 0.749	1.02, 0.24–4.40; *p* = 0.982
Hypointense vs hyperintense (ref.)	1.27, 0.38–4.25; *p* = 0.700	1.71, 0.39–7.51; *p* = 0.480
Arachnoid layer: interrupted vs intact (ref.)	2.50, 1.36–4.61; *p* = 0.003	2.44, 1.21–4.92; *p* = 0.013
Contrast enhancement: heterogeneous vs homogeneous (ref.)	2.05, 1.22–3.46; *p* = 0.007	0.91, 0.45–1.85; *p* = 0.802
Tumor shape: irregular vs regular (ref.)	2.57, 1.51–4.37; *p* = 0.001	1.76, 0.94–3.29; *p* = 0.076
Tumor calcifications: present vs absent (ref.)	0.66, 0.33–1.34; *p* = 0.250	0.59, 0.27–1.31; *p* = 0.197
Capsular contrast enhancement: present vs absent (ref.)	1.16, 0.66–2.06; *p* = 0.609	1.22, 0.59–2.51; *p* = 0.590

Subgroup analyses of 132 patients (23% of the entire collective, including 92 females, 70%, and 40 males, 40%; median age 54 years; 110 WHO grade I, 83%, and 22 grade II/III tumors, 17%) with at least 5-year follow-up after surgery confirmed disruption of the arachnoid layer (HR, 3.43; 95%CI 1.52–7.75; *p* = 0.003), heterogeneous contrast enhancement (HR, 2.16; 95%CI 1.13–4.14; *p* = 0.020), and an irregular tumor shape (HR, 2.56; 95%CI 1.31–5.00; *p* = 0.006) to be strongly correlated with progression. In multivariate analyses, GTR (HR, .26; 95%CI .09–.76; *p* = 0.013), hyperintensity on T2-weighted imaging (HR, 0.31; 95%CI 0.13–.78; *p* = 0.012), and intratumoral calcifications (HR, 0.14; 95%CI 0.04–.55; *p* = 0.005) were predictors for lower risk of recurrence, while high-grade histology (HR, 5.15; 95%CI 1.93–13.72; *p* = 0.001), and, most significantly, disruption of the arachnoid layer predicted tumor relapse (HR, 9.41; 95%CI 2.97–29.80; *p* < 0.001).

### The extent of resection is correlated with distinct imaging characteristics

In univariate analyses, GTR was less commonly achieved in skull base than in non-skull base lesions (*N* = 146, 63%, vs *N* = 272, 88%; *p* < 0.001), in tumors with an irregular than with a round, regular shape (127, 67%, vs *N* = 259, 83%; *p* < 0.001) and in lesions without than with capsular contrast enhancement (*N* = 235, 73%, vs 123, 84%; *p* = 0.013). GTR only tended to be less common in tumors with than without disruption of the arachnoid layer (*N* = 200, 73%, vs *N* = 159, 80%; *p* = 0.064). While tumor volume was not related with the extent of resection (*p* = 0.482), STR was positively correlated with edema volume (*p* < 0.001). In multivariate analyses adjusted for patients age, sex, high-grade histology, and all analyzed radiological variables, the odd of STR was higher in skull base than in non-skull base meningiomas (OR, 3.08; 95%CI 1.88–5.04; *p* < 0.001), while none of the other imaging characteristics was correlated with the extent of resection.

### MRI risk factors for progression are not congruent with predictors for high-grade histology

Comparative analyses revealed that risk factors for high-grade histology are not necessarily congruent with risk factors for progression. In fact, both heterogeneous contrast enhancement and an irregular tumor shape were associated with high-grade histology and progression. However, peritumoral edema volume correlated with grade II/III histology, while the tumor volume was associated with recurrence. Most remarkably, disruption of the arachnoid layer, the strongest predictor for progression in both uni- and multivariate analyses, was not correlated with high-grade histology (Table 4).

**Table 4:** Comparison of predictors for high-grade histology and recurrence after univariate analyses

Radiological variable	High-grade histology	Recurrence
Tumor location: Convexity/falcine vs other (ref.)	(✓)	✕
Tumor volume (in ccm)	✕	✓
Edema volume (in ccm)	✓	✕
Intensity on T2-weighted MRI	✕	✕
Isointense vs hyperintense (ref.)	✕	✕
Hypointense vs hyperintense (ref.)	✕	✕
Arachnoid layer: interrupted vs intact (ref.)	✕	✓
Contrast enhancement: heterogeneous vs homogeneous (ref.)	✓	✓
Tumor shape: irregular vs regular (ref.)	✓	✓
Tumor calcifications: present vs absent (ref.)	✕	✕
Capsular contrast enhancement: present vs absent (ref.)	✕	✕

Several risk factors were associated with both endpoints. However, tumor volume and, most remarkably, disruption of the arachnoid layer are strongly correlated with recurrence but not with histology; borderline significant correlations in brackets

✕, no correlation; ✓, significant correlation; ref., reference

## Discussion

Over the last years, a number of molecular alterations such as hTERT promoter mutations [25] or distinct DNA methylation patterns [13, 26] have been shown to improve prediction of prognosis in meningioma patients and are increasingly integrated into routine neuropathological analyses. Established predictors mostly include clinical (e.g., extent of resection [28]) and histopathological (e.g., WHO grade, Ki67 labeling index [24]) variables. On the other hand, the role of distinct characteristics on preoperative radiological imaging for prediction of prognosis remains largely unclear but could be a useful adjunct to the currently available clinical and histopathological parameters.

In a recently published systematic review, several characteristics found in routine preoperative MRI were reported to be associated with high-grade histology and/or recurrence but with partially inconsistent results [29]. Similar to findings in most of these studies, we identified an irregular tumor shape [7, 14, 16, 18, 21], heterogeneous contrast enhancement [3, 14, 16], and, with borderline significance, a non-skull base tumor location [3, 10, 12, 17, 33] to be associated with *high-grade histology*. Correlations between high-grade histology and peritumoral edema have been investigated previously with partially inconsistent results [29]. Although statistically significant, the low odds ratio and both sensitivity and specificity in our series revealed the peritumoral edema volume as a limited risk factor for histology. However, these results further underline the importance of reporting imaging findings during communication of the neurosurgeon and the neuropathologist.

Consistent with the findings in the previous smaller series, tumor volume [2, 4, 11, 21], disruption of the arachnoid layer [4, 11, 21], and an irregular tumor shape [11, 15, 16, 21] were associated with an increased risk of *recurrence*, while heterogeneous contrast enhancement was mostly not correlated with prognosis [10, 15, 16]. However, odds ratio of the tumor volume and the sensitivity and specificity were found to be low, indicating its limited value for predicting tumor recurrence. In contrast, *postoperative* residual tumor volume was shown to correlate with tumor recurrence [9]. Noteworthy, several radiological variables were found to correlate with the extent of resection. Higher rates of STR appear reasonable in skull base meningiomas or in tumors displaying a lobular growth, e.g., towards anatomical regions with limited surgical access. Similarly, distinct peritumoral edema might have altered surgical accessibility despite pre- and intraoperative administration of steroids in some cases, leading to an increased risk of STR in these patients. In contrast, association between capsular contrast enhancement and the extent of resection remains hard to explain. Moreover, variables correlated with *recurrence* were not necessarily congruent with radiological risk factors *for high-grade histology* (Table 4). Correspondingly, WHO grade-adjusted multivariate analyses confirmed an increasing tumor volume and, most noteworthy, disruption of the arachnoid layer as predictors for prognosis. This finding is particularly remarkable as disruption of the arachnoid layer was found not to correlate with the extent of resection in both uni- and multivariate analyses. In addition, long-term follow-up analyses confirmed an almost tenfold increased risk of recurrence in cases with disruption of the arachnoid layer, clearly exceeding the prognostic impact of high-grade histology.

While associations between the tumor volume and recurrence can be presumably attributed to an increased proliferative activity in these lesions, the correlation between the arachnoid layer and prognosis is difficult to explain. Associations between the integrity of the arachnoid layer and WHO grade have been investigated previously with inconclusive results [3, 7, 14, 17–19], and former analyses of our group clearly showed that disruption of the arachnoid layer on MRI does not reflect microscopical brain invasion [1]. This hypothesis is also supported by results of the present study, showing no correlations between high-grade histology and the integrity of the arachnoid layer. Similarly, previous analyses did not show correlations between loss of H3K27 trimethylation or hTERT promoter mutations and most findings on preoperative MRI including integrity of the brain/tumor surface [23], raising the question of other histopathological and molecular alterations underlying the disruption of the arachnoid layer in meningiomas. Results from Uchida et al. suggest correlations of the integrity of the arachnoid layer and contrast enhancement of the tumor capsule with the microvessel density in meningiomas [31]. Nakasu et al. reported a distinctly thinned connective tissue capsule in microscopic analyses of meningiomas with a T2-hyperintense brain/surface [20]. Associations between intratumoral calcifications and a lower risk of recurrence have been described previously and might be attributable to lower growth rates of these lesions [22]. In contrast, a lower risk of recurrence in T2-hyperintense lesions is difficult to explain and has not been described previously [10].

Although providing extensive analyses in a large series of meningioma patients, the authors are aware of some limitations of the study. Aside from the retrospective character of the series with the typical, attributed risks such as selection bias, preoperative MRI was only available in a subset of the entire patient cohort (Fig. 1) but was mostly lacking in cases being operated in the early years of the inclusion period. In fact, this might have significantly impacted follow-up, which was considerably shorter in the current patient collective than from the entire database (26 months vs 41 months, data not shown). The lack of correlation between the dichotomized extent of resection and recurrence in the entire cohort is remarkable. As subgroup analyses of patients with long-term follow-up confirmed STR as a strong risk factor for recurrence, this observation might be caused by the limited observation period of the entire study population. MRI was performed in our hospital and by a number of outpatient radiologists and other hospitals; thus, imaging quality and techniques differed widely, and technical specifications, e.g., the field strength or manufacturer, cannot be provided. Exact data about adjuvant irradiation was rarely available and was therefore not considered in further statistical calculations. Although calculated using an established formula, precision of volume quantification (especially of edemas, which might display non-spheroid spread along the white matter) is limited. On the other hand, 3D-volumetry/segmentation could not be provided due to technical reasons. An internal or external validation of the parameters on MRI has not been provided, yet. Thus, we are aware of a potential interrater variability due to subjective evaluation of the radiological variables, and therefore, an external validation is being planned in the future. Finally, data from molecular analyses and proliferation index were only available in selected patients and were therefore not considered.

In conclusion, several risk factors determinable on routine preoperative MRI for both high-grade histology and recurrence were identified, underlining the importance of considering imaging characteristics during pre- and postoperative meningioma care. Although found to be the strongest risk factor for recurrence during both short- and long-term follow-up, loss of integrity of the arachnoid layer was not correlated with histology or with the extent of resection. Thus, histological and molecular alterations underlying the disruption of the arachnoid layer in meningiomas remain to be determined.

## Electronic supplementary material

ESM 1 (DOCX 17 kb)
