# Correction to: Predicting the risk of postoperative recurrence and high-grade histology in patients with intracranial meningiomas using routine preoperative MRI

**DOI:** 10.1007/s10143-021-01630-1

**Published:** 2021-09-21

**Authors:** Dorothee Cäcilia Spille, Alborz Adeli, Peter B. Sporns, Katharina Heß, Eileen Maria Susanne Streckert, Caroline Brokinkel, Christian Mawrin, Werner Paulus, Walter Stummer, Benjamin Brokinkel

**Affiliations:** 1 Department of Neurosurgery, University Hospital Münster, Münster, Albert-Schweitzer-Campus 1, Building A1, 48149 Münster, North Rhine Westphalia Germany; 2 Institute of Clinical Radiology, University of Münster, Münster, North Rhine Westphalia Germany; 3 Institute of Neuropathology, University Hospital Münster, Münster, North Rhine Westphalia Germany; 4 Institute of Neuropathology, Otto-von-Guericke University, Magdeburg, Saxony-Anhalt Germany

**Correction to** **:** **Neurosurgical Review (2021) 44:1109–1117**

**https://doi.org/10.1007/s10143-020-01301-7**

The article “Predicting the risk of postoperative recurrence and high-grade histology in patients with intracranial meningiomas using routine preoperative MRI”, written by Dorothee Cäcilia Spille, Alborz Adeli, Peter B. Sporns, Katharina Heß, Eileen Maria Susanne Streckert, Caroline Brokinkel, Christian Mawrin, Werner Paulus, Walter Stummer, and Benjamin Brokinkel, was originally published without Open Access. After publication in volume 44, issue 2, page 1109–1117, the author decided to opt for Open Choice and to make the article an Open Access publication. Therefore, the copyright of the article has been changed to ©The Author(s) 2020 and the article is forthwith distributed under the terms of the Creative Commons Attribution 4.0 International License, which permits use, sharing, adaptation, distribution and reproduction in any medium or format, as long as you give appropriate credit to the original author(s) and the source, provide a link to the Creative Commons licence, and indicate if changes were made. The images or other third party material in this article are included in the article's Creative Commons licence, unless indicated otherwise in a credit line to the material. If material is not included in the article's Creative Commons licence and your intended use is not permitted by statutory regulation or exceeds the permitted use, you will need to obtain permission directly from the copyright holder. To view a copy of this licence, visit http://creativecommons.org/licenses/by/4.0/.

The original article has been corrected.

