# Supplementary material for: Predicting the risk of postoperative recurrence and high-grade histology in patients with intracranial meningiomas using routine preoperative MRI
Source: Neurosurg Rev. 2020 Apr 23;44(2):1109–17. doi: 10.1007/s10143-020-01301-7 (PMC8450214; doi:10.1007/s10143-020-01301-7)
Supplement: Supplementary file 1 — (DOCX 17 kb) [file 10143_2020_1301_MOESM1_ESM.docx]

| **Variable** | **Tumor volume** | **Edema volume** | **Tumor location** | **Contrast-heterogeneity** | **Capsular contrast-enhancement** | **Disruption of the arachnoid layer** | **Intensity on T2-weighted MRI** | **Tumor shape** | **Calcifications** |
| --- | --- | --- | --- | --- | --- | --- | --- | --- | --- |
| **Tumor volume** | n/a | <.001 | <.001 | <.001 | .001 | .862 | .661 | <.001 | .001 |
| **Edema volume** | <.001 | n/a | <.001 | <.001 | .015 | .714 | .847 | <.001 | <.001 |
| **Tumor location** | <.001 | <.001 | n/a | .002 | .061 | .854 | .061 | .275 | .004 |
| **Contrast-heterogeneity** | <.001 | <.001 | .002 | n/a | .280 | .027 | .002 | <.001 | <.001 |
| **Capsular contrast-enhancement** | .001 | .015 | .061 | .280 | n/a | .549 | .001 | .762 | .005 |
| **Disruption of the arachnoid l.** | .862 | .714 | .854 | .027 | .549 | n/a | .952 | .022 | .340 |
| **Intensity on T2-weighted MRI** | .661 | .847 | .061 | .002 | .001 | .952 | n/a | .603 | .121 |
| **Tumor shape** | <.001 | <.001 | .275 | <.001 | .762 | .022 | .603 | n/a | .123 |
| **Calcifications** | <.001 | .001 | .004 | <.001 | .005 | .340 | .121 | .123 | n/a |

**Supplementary table: Correlations (p-values) of the analyzed imaging characteristics.** In univariate analyses, numerous intercorrelations between the included radiological were found (cells with statistically significant p-values in grey).
